# Visions and reality: the idea of competence-oriented assessment for German medical students is not yet realised in licensing examinations

**DOI:** 10.3205/zma001102

**Published:** 2017-05-15

**Authors:** Markus Huber-Lang, Annette Palmer, Claudia Grab, Anja Boeckers, Tobias Maria Boeckers, Wolfgang Oechsner

**Affiliations:** 1 University Hospital of Ulm, Medical School, Institute for Clinical- and Experimental Trauma-Immunology, Ulm, Germany; 2 University of Ulm, Medical Faculty, Dean's Office, Ulm, Germany; 3 University of Ulm, Institute of Anatomy and Cell Biology, Ulm, Germany; 4 University Hospital of Ulm, Department of Cardiac Anaesthesiology, Ulm, Germany

**Keywords:** medical competence, high-stakes exam, alignment, roles, NKLM

## Abstract

**Objective: **Competence orientation, often based on the CanMEDS model, has become an important goal for modern curricula in medical education. The National Competence Based Catalogue of Learning Objectives for Undergraduate Medical Education (NKLM) has been adopted in Germany. However, it is currently unknown whether the vision of competence orientation has also reached the licensing examination procedures.

**Methods: **Therefore, a prospective, descriptive, single-centre, exemplary study design was applied to evaluate 4051 questions/tasks (from 28 examiners at 7 two-day licensing oral-practical exams) for undergraduate medical students at the University of Ulm. The oral and practical questions/tasks as well as the real bedside assessment were assigned to specific competence roles (NKLM section I), categories (NKLM section II) and taxonomy levels of learning domains.

**Results:** Numerous questions/tasks were set per candidate (day 1/2: 70±24/86±19 questions) in the licensing oral-practical exam. Competence roles beyond the “medical expert” were scarcely considered. Furthermore, practical and communication skills at the bedside were hardly addressed (less than 3/15 min). Strikingly, there was a significant predominance of questions with a low-level taxonomy.

**Conclusions: **The data indicate a misalignment of competence-oriented frameworks and the “real world” licensing practical-oral medical exam, which needs improvement in both evaluation and education processes.

## Zusammenfassung

**Zielsetzung: **Kompetenzorientierung basierend auf dem CanMEDS Model hat sich zu einem wichtigen Bildungsinhalt innerhalb der medizinischen Ausbildung entwickelt. Im Nationalen kompetenzbasierten Lernzielkatalog Medizin (NKLM) wurde diese als Ziel festgeschrieben. Es ist jedoch bisher nicht bekannt, inwiefern in den Prüfungen des medizinischen Staatsexamens tatsächlich kompetenzbasierend geprüft wird.

**Methoden:** In dieser prospektiven, deskriptiven, monozentrischen, exemplarischen Studie wurden 4051 Fragen/Aufgaben (gestellt von 28 Prüfer in n=7 zweitägigen mündlich-praktischen Prüfungen) an Medizinstudierende der Universität Ulm evaluiert. Die mündlichen und praktischen Fragen/Aufgaben wurden, ebenso wie die Prüfung am Patientenbett, auf die spezifischen Kompetenzrollen (gemäß NKLM Abschnitt I), die unterschiedlichen Kategorien (gemäß NKLM Abschnitt II) und die Taxonomielevel der unterschiedlichen Fragen/Aufgaben analysiert.

**Ergebnisse: **Im mündlich-praktischen Prüfungsteil wurden den Kandidaten zahlreiche Fragen/Aufgaben gestellt (Tag 1/2: 70±24/86±19 Fragen). Die Kompetenzrollen außerhalb des “Medizinischen Experten” wurden dabei selten adressiert. Zudem wurden praktische und kommunikative Fertigkeiten in der Prüfung am Patientenbett kaum geprüft (weniger als 3/15 min). Auffallend war die Dominanz an Fragen auf niedrigem taxonomischem Niveau.

**Schlussfolgerungen:** Die Daten zeigen ein deutliches Missverhältnis zwischen den Vorgaben zur Kompetenzorientierung und den tatsächlich geprüften Inhalten der mündlich-praktischen Prüfungen. Diese Diskrepanz sollte zukünftig mit einer verstärkten Prüfer-Schulung und -Evaluation begegnet werden.

## 1. Introduction

### 1.1. Background

The classical concept of learning-objective orientated education, which over many decades has represented the conceptual basis for higher education, has been increasingly broadened by a much more complex concept of competence based education. Since their first publication in the 1990s, the 7 CanMed roles (medical expert, communicator, collaborator, manager, health advocate, scholar, and professional) have served worlwide as the “archetype” for medical competence orientation [1], [2], [3]. With reference to the CanMEDS framework, the National Competence-Based Learning Objectives for Undergraduate Medical Education (NKLM) has been recently introduced throughout Germany as a framework for competence-based medical education on the basis of the national licence order [4], [5].

To assess the grade of clinical competence finally attained by medical students, oral-practical examinations have been used as a major instrument for decades [6], [7], [8], [9]. In general, classical oral-practical exams remain subject to serious criticism based not only on low-rated validity and reliability [6], [10], [11], but also on difficulties in addressing the whole spectrum of “medical competences”. In contrast, positive examples for well-structured and competence oriented oral examinations are only occasionally described [12].

Currently, there are no direct observation data of the licensing oral-practical examinations in Germany available in regard to the degree of their competence orientation and to the taxonomy level of the questions and tasks used to assess the students. 

#### 1.2. The German Licensing Exam

Though recently changed, at the time period of our study the licensing exam for medicine in Germany consisted of two sections. Both of them, section I after 2 years and section II after 6 years, contained a written and an oral / oral-practical part. The change recently performed associated the written part of the exam section II to an earlier study phase (after 5 years) and declared the oral-practical part as exam section III after 6 years. However, the actual process of the oral-practical part, lasting two days, has not been affected by this change. The concrete details of the examination process as described in the following article are, within the framework of the German National License Order of Germany, regulated by the responsible State Examination Office of Baden-Wurttemberg. 

**Figure 1:**
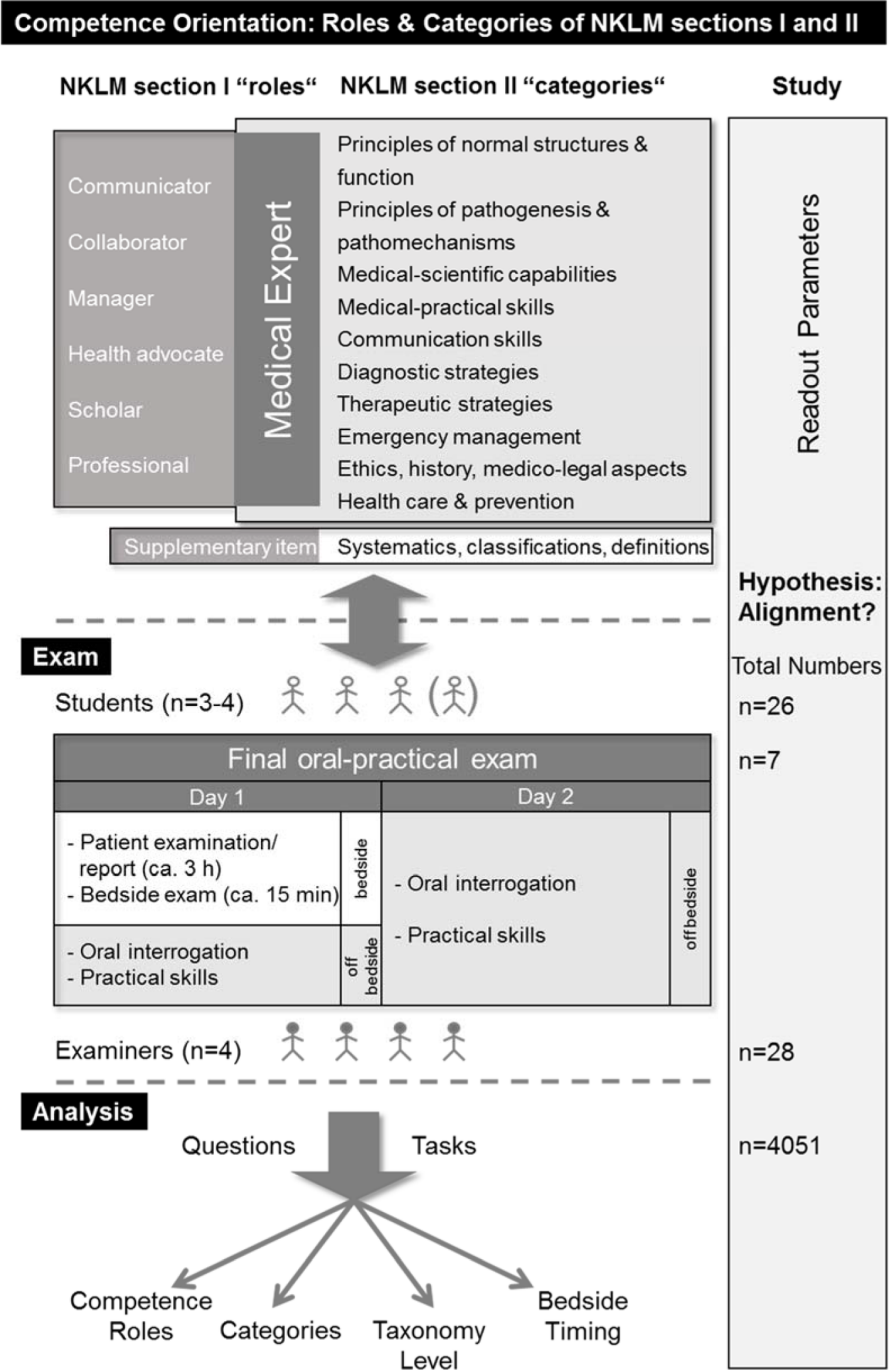
Competence Orientation: Roles & Categories of NKLM sections I and II

During the two days of the oral-practical examination part, the examiners are given an ample scope within the regulatory framework in regard to the content of the questions and tasks they plan to set the candidates [13], [14]. On day 1, after a 3-h time slot for each candidate to investigate and document a real patient’s case at the bedside, the candidate (one of up to four students) has approximately 15 minutes to present the patient’s case to the three examiners, to demonstrate examination techniques (on the examiners’ demand) and to answer patient-oriented questions (see Figure 1 (Fig. 1)). Off-bedside, an interrogation of each candidate follows (7-11 minutes per examiner). On day 2, each candidate is interrogated by each examiner for 11-15 minutes. The examination is performed by the same four examiners, who are permitted to ask questions or to set tasks from any discipline. 

Each student is finally scored on a scale of 1–5, having successfully passed the oral-practical part of the exam if a score ≤4 is achieved.

Having successfully passed all examination parts, the candidates may apply for the full licence to practise medicine (approbation) in Germany and in the European Union, with the licence being valid for an unlimited time. 

In the present prospective, exemplarily performed single-centre study, we investigated to which degree medical competences other than the traditional “medical expert” are represented in the German licensing oral-practical exam. Furthermore, we explored the taxonomy levels (in approximation to Bloom’s taxonomy of learning domains [15]) of the questions and tasks adressed during this important examination.

## 2. Methods

### 2.1. Study design

The present analyses were based on a prospective, observational-descriptive, single-centred, exemplary study design. Approval was obtained from the local independent Ethics Committee of the University of Ulm (17122013) and from the dean’s office. A pilot study with observation of two licensing oral-practical exams tested the feasibility and practicability of the observation tools. The experience of the pilot lead to the hypotheses mentioned above. The main study then included the observation of days 1 and 2 of n=7 exams (during the winter semester 2013/14 and summer semester 2014). In accordance with the nationwide medico-legal framework (AeAppO 2002/2012), one study observer (not involved as an examiner) was present per oral-practical exam. The overall two observers met the following criteria: they were members of the faculty teaching body, have been actively involved in academic teaching and evaluation processes for more than 10 years, functioned as trainers in examiner training for more than 5 years, completed a master of medical education (MME) study-course, have been acquainted with NKLM criteria, and have been members of the university’s task force for research in medical education. Two observers were intensively pre-trained based on the developed observation tools. The inter-rater reliability (Cohen´s Kappa) between the two evaluators was calculated based on the analysis of 50 questions/tasks from real examinations according to both the assessment of the taxonomy levels (in approximation to the criteria described by Bloom 1956 [15]) and the attribution to various competence roles and content categories/subjects [http://www.nklm.de accessed:13 Oct 2015]. A combined inter-rater reliability of 0.78 was considered sufficient for the study. However, if any question could not be classified onsite, the exact wording of the question was documented and later clarified between the two observers to reach a consensus (which occurred in 20 cases). 

#### 2.2. Observation and Evaluation Instruments

##### 2.2.1. Bedside exam document 

For data acquisition during the beside exam, one document has been designed to record the measured time as well as the number of questions/tasks and their assignment to the respective taxonomy level. 

##### 2.2.2. Off-bedside exam document day 1 and 2 

For data acquisition during the off-bedside examination, an one-page document has been developed, which allows categorisation of each individual question/task (of each examiner) for different categories/subjects and taxonomy levels, inclusive of open ended questions for the observer’s comments. All the described documents were tested for feasibility and practicability in a pilot study (during two full exams), and consequently slightly adjusted. 

#### 2.3. Chronometry of patient-contact during the oral-practical bedside exam

To assess the utilisation of the examination time for direct patient-examinee-communication, -contact, -interaction, and -practical-skill demonstration, the respective time was recorded in seconds using a conventional stopwatch and documented. The *post hoc* analysis then added the timing in relation to the total bedside examination time.

#### 2.4. Assessment of the licensing oral-practical exam regarding competence-oriented issues

After each exam the data were transferred by an independent person to a digital database and – after complete data gathering – analysed regarding the following read-out parameters:

- Number of questions/tasks (per examiner, examinee, time slot)
- Taxonomy level of questions/tasks. In approximation to the Bloom’s classification [15], a simplified three-levelled attribution was performed for the medical questions and tasks, with “knowledge” (in the sense of pure reproduction of factual knowledge) as the first and lowest taxonomy level, “analysis/interpretation” as the second, intermediate level, and “problem solving” as the third level. The pilot study confirmed the feasibility of this simplified 3-step categorisation during the onsite situation of the examination.
- Assignment, if suitable, of questions/tasks to one of the competence roles apart from the central “Medical Expert” role (in accordance to CanMEDS and NKLM): Communicator, Collaborator, Manager, Health Advocate, Scholar, Professional.
- Assignment of the questions/tasks to various content categories in approximation to the NKLM section II (see Figure 1 (Fig. 1)). The pilot study revealed numerous questions addressing “systematics, classifications, and definitions”; therefore, we added this category as an additional read-out parameter.

#### 2.5. Statistics:

The present analyses are based on a prospective, observational-descriptive, single-centred, exemplary study design. Results are presented as the mean ± standard error of the mean (SEM). A one-way analysis of variance (ANOVA) followed by the Student-Newman-Keuls test as a post hoc test for multiple comparisons was performed. A comparison of read-outs between exam days 1 and 2 was performed by the Student-t test. A value of *p*<0.05 was considered statistically significant.

## 3. Results

### 3.1. A considerable number of questions and tasks are demanded in the licensing oral-practical medical examination

In present study of seven licensing oral-practical medical exams (each of 2-day duration) with n=28 examiners (4 in each exam) and n=26 examinees (3-4 in each exam), a total of n=4051 raised questions/tasks were analysed. During the first day, n=70±24 questions/tasks were set per candidate and during the second day n=86±19 questions/tasks were allotted per candidate (data not shown).

#### 3.2. Minimal address of practical and communication skills by examiners at the bedside during the licensing medical exam

**Figure 2:**
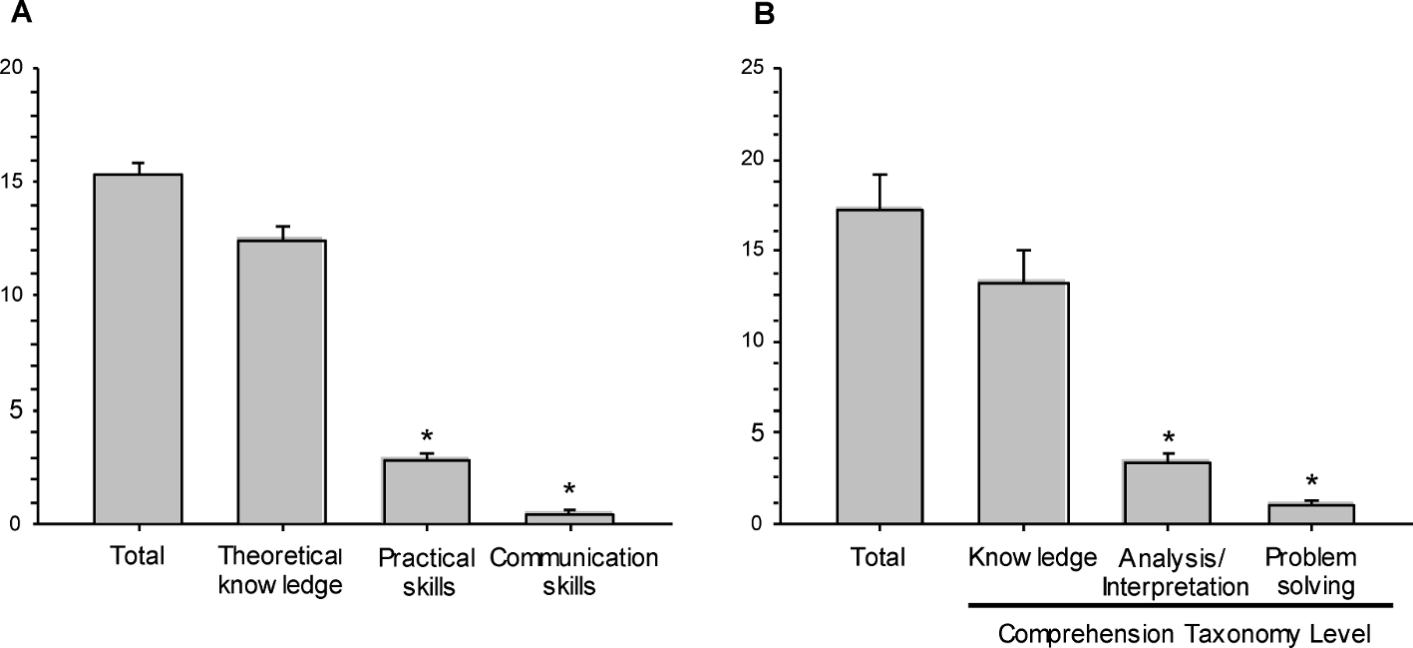
Bedside practical-oral exam A Time dedicated to theoretical knowledge, practical or communication skills; *p<0.05 vs. theoretical knowledge. B Taxonomy level of questions; Number of questions during the exam addressing three taxonomy levels: knowledge, analysis/interpretation and problem solving. *p<0.05 vs. knowledge; data are presented as mean ± SEM; (One-Way-Anova, n=26 students/n=28 examiners)

The bedside presentation of the patient, including the main findings and the demonstration of specific practical and communication skills on demand, is scheduled (according to the State Examination Office of Baden-Wurttemberg) for a time period of regularly 15 minutes. The scheduled total bedside exam time was perfectly adhered to by the examiners (see Figure 2 (Fig. 2), Point A). However, during this bedside time, >80% of exam time was used to ask and to answer theoretical questions, <20% of the exam time was used to demonstrate hands-on practical skills and virtually no communication skills were explicitly addressed. The student was challenged with >17±9 (theoretical) questions at the bedside (i.e. >1 question per minute). Furthermore, the analysis of the taxonomy level of these questions revealed that 13±2 targeted a low taxonomy level (i.e. the “knowledge” level; see Figure 2 (Fig. 2), Point B), 3±0.1 (18%) bedside questions considered the analysis or interpretation level and one question was based on the level of problem solving. 

#### 3.3. Competence roles beyond the “medical expert” are scarcely reflected in the licensing oral-practical medical exam 

**Figure 3:**
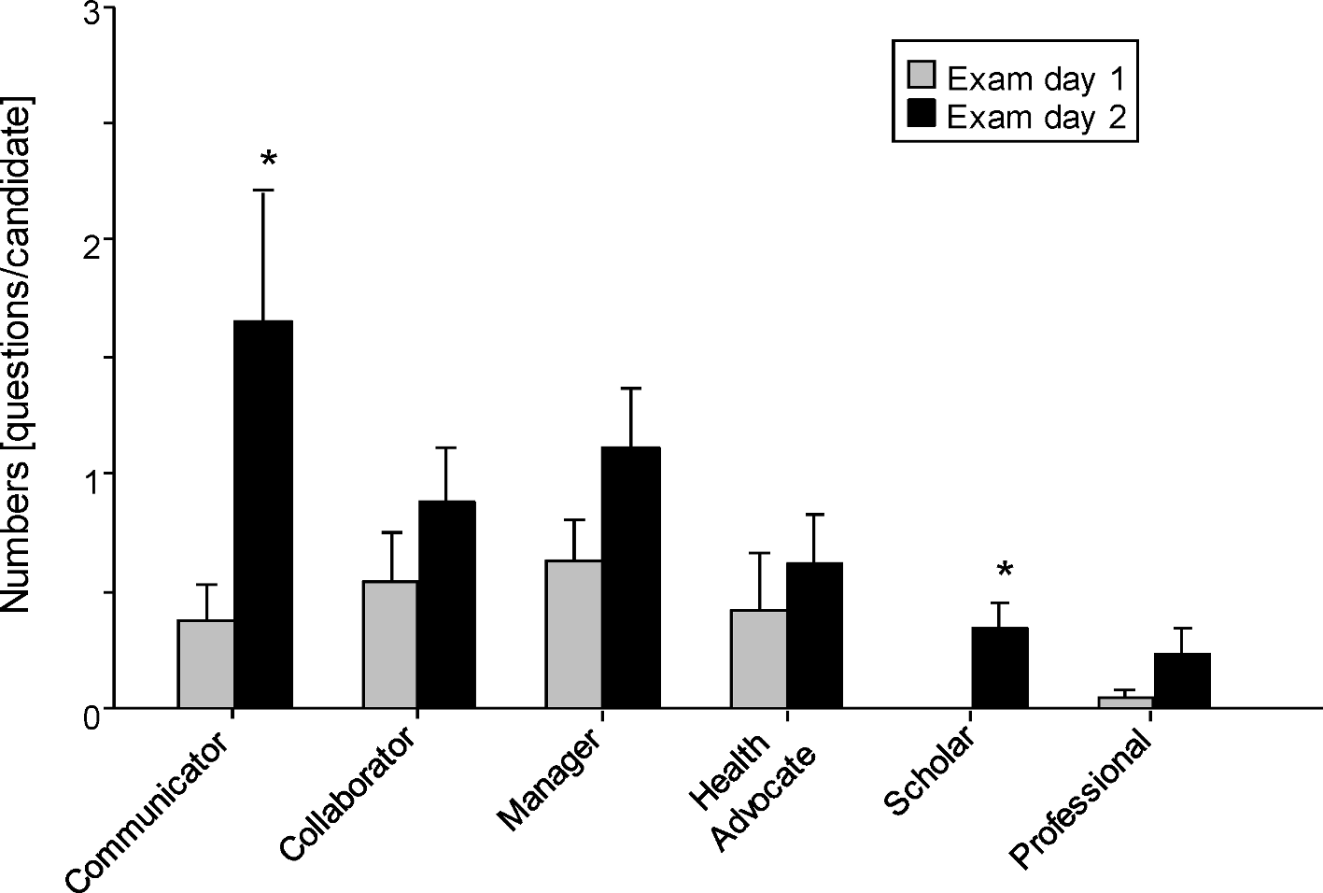
Off bedside practical-oral exam: Addressed roles Number of questions per candidate addressing the different roles on both days of the exam; data are presented as mean ± SEM; *p<0.05 vs. number of questions at day one (t-test).

The NKLM section I framework, derived from the CanMEDS Physician Competence Framework [1], is to some extent based on the seven roles as a prerequisite for all physicians for good and reliable patient care: Medical Expert, Communicator, Collaborator, Manager, Health Advocate, Scholar, and Professional. The “medical expert”, considered as the classical and traditional role, and standing “in the centre” of all roles, certainly plays the main role in teaching, learning, and assessing medicine. Therefore, we were mainly interested in learning to what degree the six roles, other than the “medical expert”, are represented as examination subjects. The present analysis revealed that on both examination days a mean of <1 (maximal 2) question representing competences of a role beyond the medical expert was put to each candidate (see Figure 3 (Fig. 3)), and that virtually no question addressed the role of the professional or scholar. 

#### 3.4. The licensing oral-practical exam focuses on very “traditional” subjects and categories

Professional expertise for the competent physician comprises a range of items and subjects from various categories, including knowledge, skills, and attitudes, which section II of the NKLM attempts to define (see Figure 1 (Fig. 1)). 

**Figure 4:**
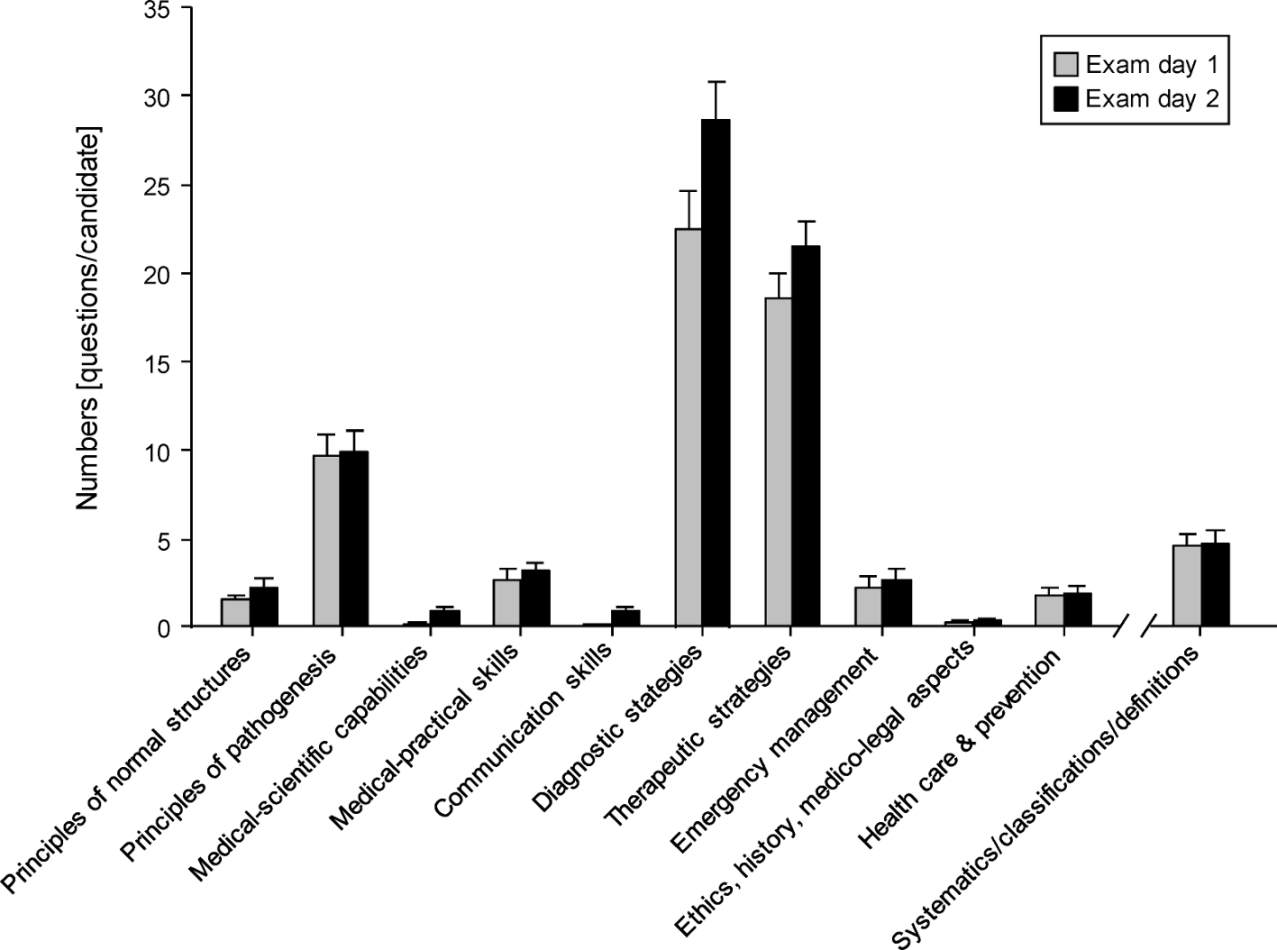
Off bedside practical-oral exam: Addressed topics Number of questions regarding the different categories per candidate on both days of the exam; data are presented as mean ± SEM; No statistically significant differences (p<0,05) between the number of questions of day one and two (t-test).

Each question or task of the two day examination was classified in one of the 11 content categories of the observation tool. The main subjects were associated (in progressional order) with questions to the “classical” or “traditional” line of systematics/classifications/definitions, pathomechanisms, therapy, and finally diagnostics (see Figure 4 (Fig. 4)). Only a minority of the questions/tasks were aimed at emergency management issues (together 5±4.5 questions on both days of the exam) and almost none at medico-legal and ethical aspects or health care and prevention. In addition, a mean of <1 question per candidate was raised both regarding scientific issues and in the context of medical conversation/counselling techniques (communication skills). The assessment of clinical and practical skills, although permitted by the legal examination framework, played only a minor role. 

#### 3.5. Predominance of questions with a low-level taxonomy 

**Figure 5:**
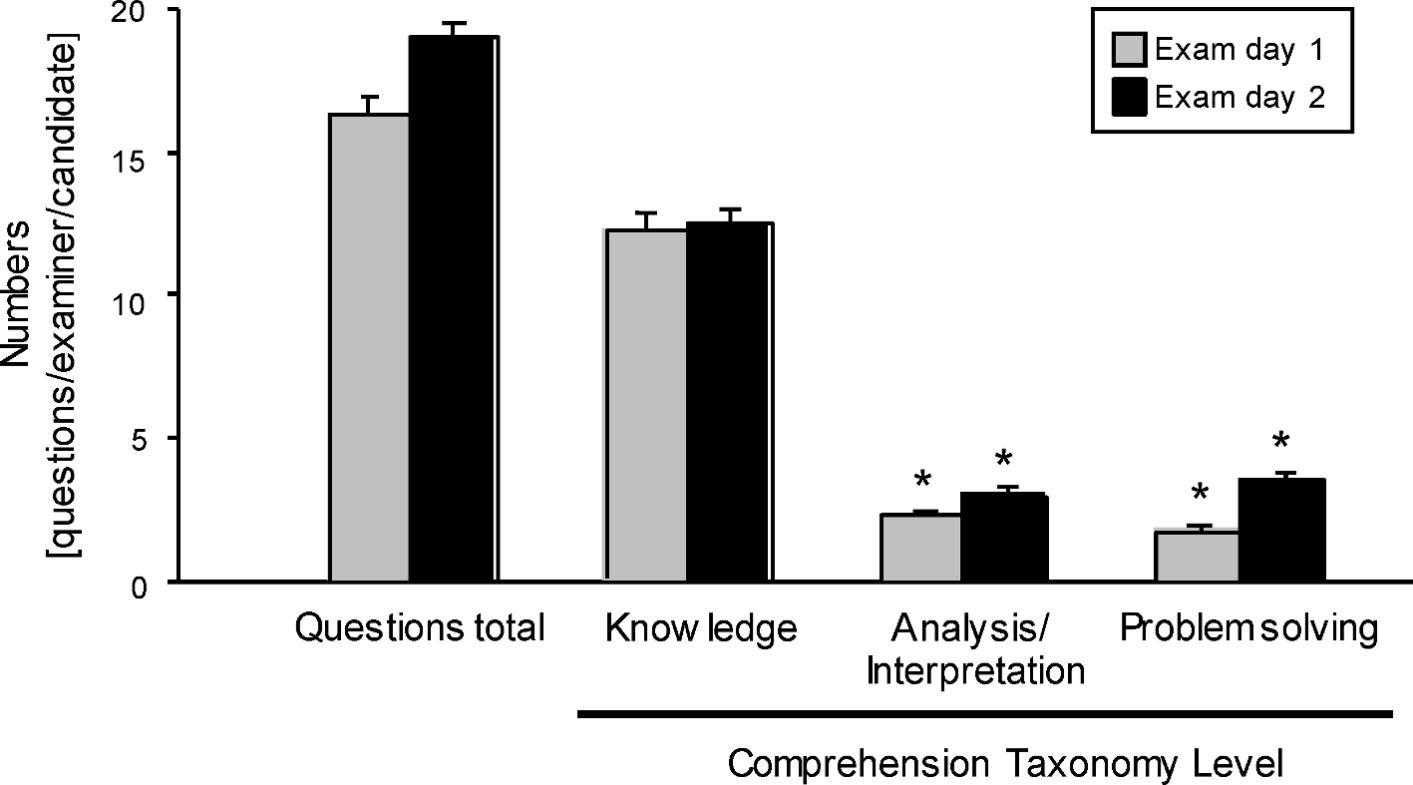
Off bedside practical-oral exam: Taxonomy level of questions Number of questions per examiner and candidate attributed to three taxonomy levels: knowledge, analysis/interpretation and problem solving on both days of the exam. Data are presented as mean ± SEM; *p<0.05 vs. knowledge (One-Way-Anova).

All the questions raised were categorised by the observers within three taxonomy levels, namely “knowledge”, “analysis/interpretation”, and “problem solving”. The majority of the questions (70% on both days) were categorised as low-level, pure “knowledge” questions. Less than 15% of the tasks were evaluated as mid-level tasks requiring some analytical and/or interpretative competence. Similarly, the level of “problem solving” was addressed in <10% on day 1 and <20% on day 2. Overall, the taxonomy level of each examiner’s questions per candidate did not significantly differ between the first and second days of the licensing exam (see Figure 5 (Fig. 5)).

**Figure 6:**
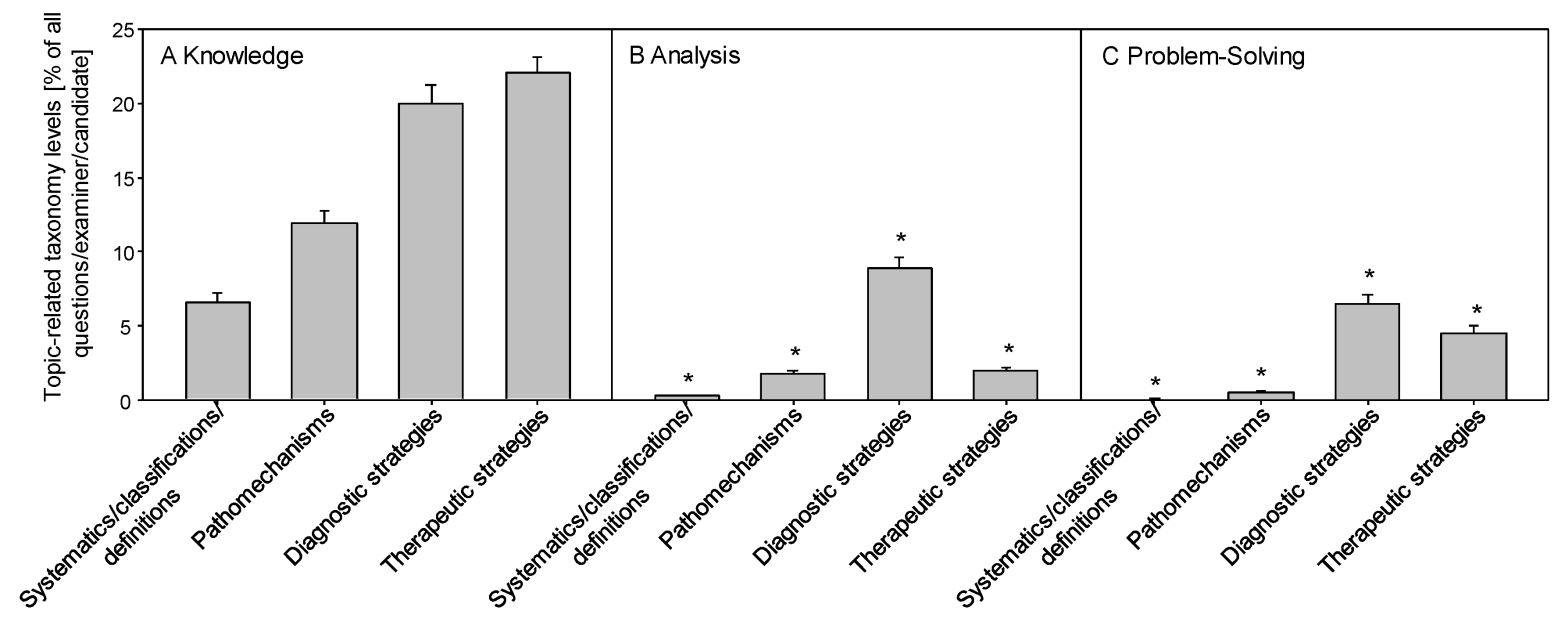
Off bedside practical-oral exam: Topic specific taxonomy levels Percentage of questions regarding the different categories per examiner and candidate on both days of the exam assessed by three taxonomy levels: (A) knowledge, (B) analysis/interpretation and (C) problem solving. Data are presented as mean ± SEM; *p<0.05 vs. taxonomy level “knowledge“(One-Way-Anova)

A more detailed analysis of the taxonomy levels in relation to the mainly examined expertise subjects or categories (>3 questions/subject) shows that the examination was dominated by “traditional” categories and by low-level (knowledge) questions (see Figure 6 (Fig. 6)). Even the category “therapeutic strategies” failed to induce questions of higher taxonomy levels such as analytical, interpretative and problem solving competences. 

## 4. Discussion

Based on an etymological analysis, the word “examine” originates from the Latin “examinare” meaning "accurately weighing" [16]. To exactly “weigh” the performance profile of a candidate in a high-stakes medical exam, an assessment of multiple medical and personal competencies is required [6], [17], [18], [19]. Theoretically, the German licensing examination offers the chance to “weigh the candidates’ performance” by a written 2-day multiple-choice-test and a 2-day oral-practical examination, each format aiming towards different professional requirements and competences [20]. 

However, the present study indicates a clear gap between the demanded competence-orientation in medical education (that is generally rated as highly relevant by clinical teachers and examiners) [21], [22] and the real-life situation during the oral-practical part of the investigated licensing medical exam (which tends to represent an assessment of pure knowledge within a single competence role). In concrete terms, the physician’s roles beyond the “medical expert” role were rarely addressed by the examiners, both at the bedside and in the examination room. Even the “communicator” role, which could have been addressed relatively easily during the bedside exam, was, in fact, very rarely addressed, although it is, without any doubt, of uppermost importance in a physician’s daily work. The competence of communicating with patients and their families has, to some extent, a predictive character regarding the frequency of future legal disputes between patients and physicians [23]. Furthermore, virtually no questions or tasks were related to the roles of the “professional”, “health advocate”, or “scholar”. Again, similar to the “communicator” role, the neglect of the “scholar” role was also somewhat surprising, because all investigated examiners had – in addition to their clinical expertise – a profound academic/scientific background and all of them were considered to be scientifically active, such that the “scholar” role was supposed to play a significant role in their daily academic work. Additionally, from the perspective of the students, scientific education appears important as reflected by a generally positive attitude towards science and scientific methodology [24]. The omission of scientific/scholar aspects by the examiners may also be considered “alarming”, because mastering scientific algorithms reflects unique academic competence with a high impact in medical decision making, self-criticism, and scientific evidence-based case-reflection that may even improve patient safety [25]. In this context it is noteworthy that the NKLM does not predefine a specific weighting of the different roles and competences. This is certainly an important issue for future discussions among medical educators. 

However, even within the singular role of the “medical expert” that has been mainly addressed by the examiners, there is surely more than pure knowledge that could be assessed: Miller’s pyramid proposes “Knows” – “Knows how” – “Shows how” – “Does” as different taxonomic levels, with the higher levels perfectly suited for oral-practical exams. Additionally, within the framework of the current licensing exam, an ideal opportunity is foreseen to make use of higher taxonomic levels: the bedside exam of examination day 1, which is obligatory throughout Germany and which is (by the State Examination Office of Baden-Wurttemberg) recommended to take at least 15 minutes per candidate. During this time slot, one would expect the clinical experts to evaluate the candidates’ competence with respect to patient interview and physical examination. However, our analysis of the bedside exam revealed a “question storm” occurring at the bedside, mostly questions of a low-level taxonomy that could have easily been asked in the later phase of the oral exam offside the patient’s bed. 

During the second exam day, no patients are involved, but the legal framework nevertheless provides multiple degrees of freedom for the examiners to assess the medical competences of the candidates in a multi-perspective approach, be it with the help of 3-D models or manikins and by imagined scenarios of medical dilemmas. However, the results of our analysis do not differ from the findings of the first day: roles besides the “medical expert” and subjects apart from the classical items of “diagnosis and therapy” were almost completely omitted. 

The most likely explanation for this phenomenon is that the examiners’ awareness and ability of assessing these roles and subjects are not yet sufficiently developed. 

Both deficiencies could potentially be remedied when in future in the training of examiners greater focus is consciously placed on the importance of these roles for the overall medical competence and to provide training for appropriate examination techniques [9], [18], [26]. 

Nevertheless, examiner trainings (offered at many faculties) are naturally attended by predominantly novice examiners. Already active and experienced examiners are far less attainable for specific examiner trainings. At this point, all measures for faculty and human resources development should therefore be exploited in teaching and examination alike, to constantly make teachers and examiners at the faculties aware of the importance and the added value of competence orientation, and to apply the appropriate tools to implement this [19], [20], [27], [28], [29]. 

In this regard, the German License Order (Aerztliche Approbationsordnung) unfortunately at present even places certain constraints on further developments. Although it allows creative scope for competence orientation in teaching, the licence order makes no binding requirements in the direction of competence-oriented testing. In addition, more specific competence based exam formats for the licensing exams are currently not explicitly planned – such as an OSCE, which in the meantime has become established in the clinical intermediate exam and in the Swiss Federal Licensing Examination, or of the SOEs (Structured Oral Examinations with predefined clinical scenarios) described by Jefferies et al. [2], [12], [30], [31], [32], [33]. 

To our knowledge, the present study is the first onsite real-time analysis of the licensing oral-practical medical exam in Germany. However, there are some drawbacks and limitations of which to be aware. The data were obtained at only one centre and therefore translation to other nationwide exam sites may only acceptable if further centres are included. Although an acceptable inter-rater ratio was achieved, further observers or recording of exams and post hoc blinded analysis would enhance the validity and reliability of the data. However, in the present setting, the given medico-legal framework prevented us including more observers or to tape the exam for analytic purposes. Furthermore, live-coding of the exam also lead to restriction on explicit aspects of the examination questions with deliberate negligence of all implicit aspects. It should be emphasized, that although the idea of competence orientation was considered long before publication of the NKLM in Germany, the concrete content of the NKLM catalogue in its actual form has only recently been finalised and accepted by the authorities, and therefore requires time to be transferred to the educational curricula and various disciplines [4], [34]. At present, the NKLM has set the goals; the constructive alignment demands now subsequent development of both teaching and assessment systems. For the future of the German licensing examination system, the NKLM offers an enormous potential to push it towards a better competence orientation.

## 5. Conclusions

In conclusion, the present study revealed a severe misalignment between proposed competences and learning objectives (as defined in the NKLM and CanMEDS) and the learning objectives assessed in current real-life high-stakes examinations. The investigated exams followed a rather simple and traditional line, with a large number of questions on a low taxonomy level, with only minor practical parts. 

Following the well-known paradigm “assessment drives learning” the multi-dimensional “competencies” aspect needs to be integrated in these oral-practical exams and addressed by the respective curricula. These changes presumably may also improve patient care and safety in the long term [35], [36].

## Acknowledgements

The authors thank Stephanie Denk for graphical support. We especially thank Prof. Dr. med Jana Juenger (University of Heidelberg) for her excellent assistance in the modification of the study design. We also want to express our gratitude to Dr. Robert Blakytny for cross reading the manuscript and correcting the language. 

## List of abbreviations

- ANOVA: Analysis of variance
- CanMEDS: Canadian Medical Education Directives for Specialists
- MME: Master of Medical Education
- NKLM: National Competence-based Learning objectives for undergraduate Medical education
- OSCE: Objective-Structured Clinical Examination
- SEM: Standard Error of the Mean

## Ethics approval and consent to participate

Approval was obtained from the local independent Ethics Committee of the University of Ulm (17122013).

## Availability of data and materials

Data are stored at the Department of Orthopaedic Trauma, Hand-, Plastic- and Reconstructive Surgery, University Hospital of Ulm, 89081 Ulm, Germany. Data are not yet shared because the extensive excel files includes additional data that are not focus of this publication.

## Competing interests

The authors declare that they have no competing interests.
